# Integrating bioinformatics and molecular experiments to reveal the critical role of the cellular energy metabolism-related marker PLA2G1B in COPD epithelial cells

**DOI:** 10.3389/fimmu.2025.1666195

**Published:** 2025-10-16

**Authors:** Jun Shi, Zihan Wang, Yafei Rao, Danyang Li, Ying Luo, Yue Zhang, Yuqiang Pei, Xiaoyan Gai, Yongchang Sun

**Affiliations:** Department of Respiratory and Critical Care Medicine, Research Center for Chronic Airway Diseases, Peking University Third Hospital, Peking University Health Science Center, Beijing, China

**Keywords:** chronic obstructive pulmonary disease, cellular energy metabolism, machine learning, PLA2G1B, single cell sequencing

## Abstract

**Background:**

Chronic obstructive pulmonary disease (COPD) is a chronic respiratory disease characterized by small airway lesions and persistent airflow limitation. Recent studies have highlighted impaired cellular energy metabolism (CEM) in COPD, although the underlying mechanisms remain incompletely understood.

**Material and methods:**

This research identified cell energy metabolism-related differentially expressed genes (CEM-DEGs) by collecting CEM-associated signatures from multiple public databases and integrating these markers with data from the GEO database. Subsequently, five machine learning algorithms—Boruta, Xgboost, GBM, SVM-RFE, and LASSO—were employed to screen for key variables. Gene Set Enrichment Analysis (GSEA) and immune infiltration analysis were then performed on these key CEM-DEGs. Finally, the results of the bioinformatics analysis were verified by *in vitro* and *in vivo* experiments in combination with the single-cell data analysis results.

**Results:**

Bioinformatic analysis identified six critical markers (*CYP1B1*, *CA3*, *AHRR*, *MGAM*, *PNMT*, and *PLA2G1B*) that regulated CEM in the progression of COPD, from which a prognostic model was constructed using a nomogram with an area under the curve (AUC) of 0.814. Functional enrichment analysis further elucidated the intricate interplay between these CEM regulatory factors and key biological processes, including inflammation, oxidative stress, and epithelial-mesenchymal transition. Beyond that, both *in vitro* and *in vivo* experiments, along with single-cell data analysis, have conclusively verified the specific downregulation of *PLA2G1B* in epithelial cells derived from the COPD group. Notably, the knockdown of *PLA2G1B* in epithelial cells triggered inflammation, oxidative stress, and apoptosis.

**Conclusions:**

This study identified six CEM-related biomarkers (*CYP1B1*, *CA3*, *AHRR*, *MGAM*, *PNMT*, and *PLA2G1B*) in COPD and established a corresponding prognostic model. Furthermore, *in vitro* and *in vivo* experiments validated the regulatory role of PLA2G1B in epithelial cell inflammation, oxidative stress, and apoptosis, thereby elucidating the mechanism underlying CEM in COPD and potentially uncovering novel therapeutic targets for drug development.

## 1 Introduction

Chronic obstructive pulmonary disease (COPD) is a chronic inflammatory airway disorder characterized by irreversible airflow limitation, primarily associated with long-term cigarette smoking and genetic predisposition. Currently, it is one of the top five age-standardized causes of death worldwide, imposing a heavy burden on healthcare and the economy (1, 2). The main pathophysiological features of COPD include chronic inflammation, airway remodeling, and emphysema, which can lead to clinical manifestations such as dyspnea, cough and expectoration in patients (3). The chronic inflammatory response of COPD involves the participation of various inflammatory cells, including immune cells (neutrophils, eosinophils, macrophages, T cells and mast cells) and lung structural cells (epithelial cells, fibroblasts and endothelial cells) (4). Exogenous stimuli and injuries induce inflammatory cells to release a large number of inflammatory factors and various proteases, triggering pulmonary inflammatory responses and damaging lung structure. At the same time, the chemokines released by these inflammatory cells recruit more inflammatory cells from the blood circulation, further intensifying the inflammatory response and leading to lung tissue damage, destruction of alveolar structure, and airway remodeling (4, 5).

Recent studies have revealed significant abnormalities in metabolic energy changes in the plasma and lung tissues of patients with chronic obstructive pulmonary disease (COPD), as these altered metabolic products are crucial risk factors that disrupt the normal energy supply in the body, suggesting that interventions targeting these metabolic-related markers may emerge as a promising new strategy for treating COPD (6–8). For example, COPD patients showed abnormal changes in lipid metabolism, especially in fatty acids and acylcarnitines, and these differentially expressed lipid metabolites could be used to accurately diagnose the occurrence of COPD (9). Ren et al. also revealed abnormal changes in amino acid metabolism in the serum of individuals at the pre-COPD stage (10). At the cellular level, the inhalation of cigarette smoke stimulates airway epithelial cells, alveolar epithelial cells, vascular endothelial cells, and alveolar macrophages within the tissues. This disruption affects various aspects of cellular energy metabolism (CEM), including lipid, glucose, and amino acid metabolism, ultimately leading to a shortened cell lifespan and an acceleration of disease progression (6, 11, 12). The latest research indicated that alveolar macrophages communicated with distant vascular endothelial cells by releasing ceramide-containing vesicles. Inhibiting the expression of enzymes related to *de novo* ceramide synthesis in alveolar macrophages effectively prevented the destruction of the endothelial barrier, suggesting that the intrinsic energy metabolism of cells may even affect distant cell communication (13). Beyond that, previous studies have also found that targeting pyruvate-citrate metabolism in airway basal cells (14), GSH metabolism in airway epithelial cells (15), and glycolysis in macrophages (16) are all effective therapeutic targets for COPD. In summary, current research has discovered a close association between COPD and CEM, but there is still a lack of comprehensive understanding of the specific molecular mechanisms underlying CEM disturbances in lung cells.

**Figure 1:**
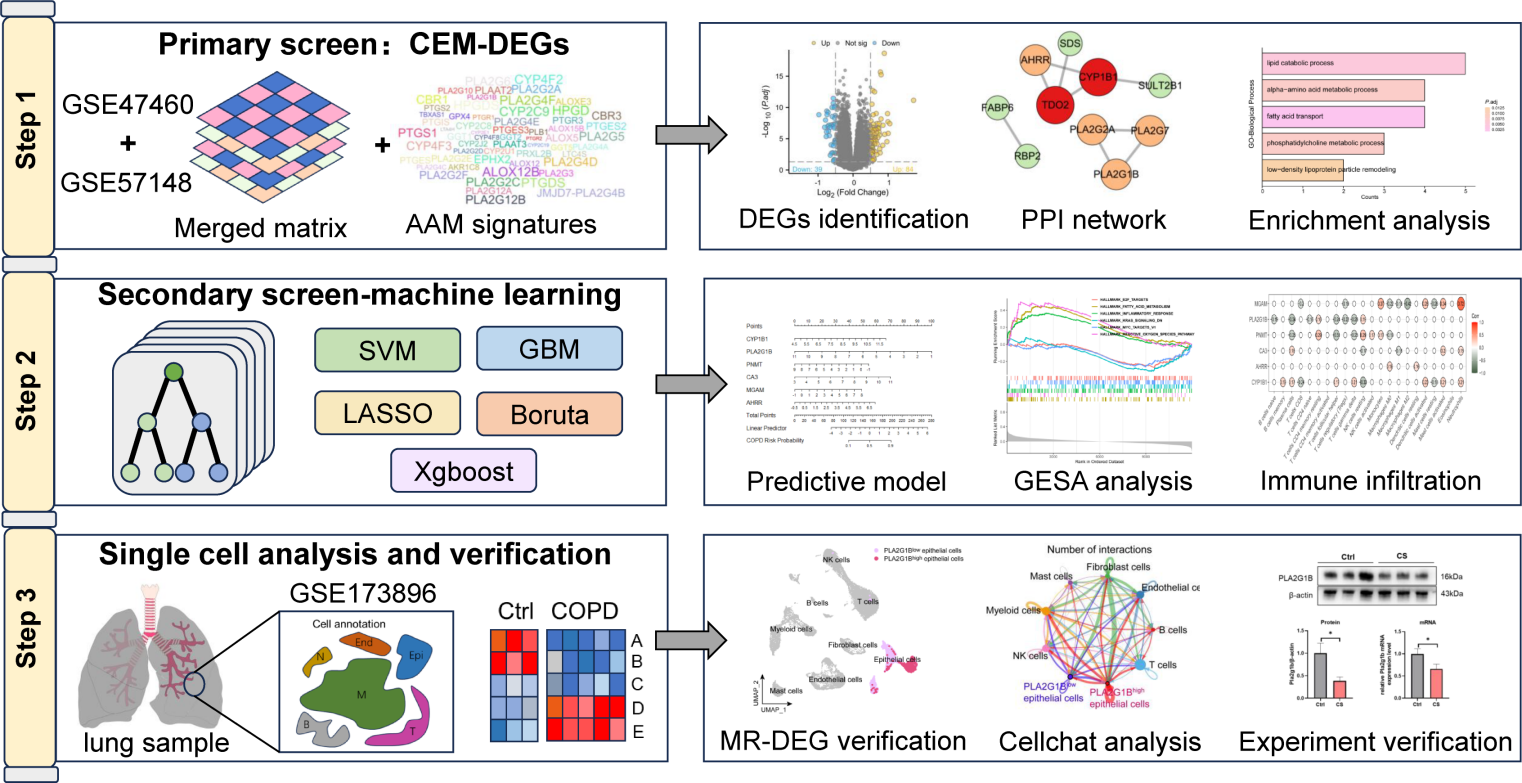
The workflow diagram of this study showed.

Our study aims to identify and validate biomarkers related to CEM during the progression of COPD through bioinformatics analysis combined with molecular biology experiments, thereby providing new insights into the pathogenesis of COPD and new strategies for its treatment. **Figure 1** showed the workflow of this study. By integrating the largest current COPD transcriptome dataset, differentially expressed genes (DEGs) related to metabolic dysregulation were identified. Next, five machine learning algorithms were utilized to further identify and screen the key variables, followed by the construction of a prognostic model based on the screened variables. In addition, single-cell data analysis confirmed the key role of phospholipase A2 group IB (PLA2G1B) in epithelial cells, and molecular biology experiments demonstrated that knockdown of PLA2G1B aggravated inflammation, oxidative stress, and apoptosis in epithelial cells.

## 2 Materials and methods

### 2.1 Bioinformatics analysis

#### 2.1.1 Identification of CEM-related DEGs

The transcriptome sequencing datasets associated with COPD (species: Homo sapiens, sample type: lung tissue) were retrieved from the GEO database (https://www.ncbi.nlm.nih.gov/geo/). Among these, two datasets with the largest sample sizes were selected for further analysis: GSE47460 (17), which includes a total of 328 samples (220 COPD cases and 108 normal controls), and GSE57148 (18), which comprises 189 samples (98 COPD cases and 91 normal controls) (**Supplementary Table S1**). Based on the R platform (version 4.3.2) the “sva” package was used to further eliminate the batch effect from the two datasets and merge them into a comprehensive large-scale data matrix. Then, the “limma” package was further used to identify COPD-related DEGs with |log2 fold change (FC)| > 0.5 and adjusted p-value (*p*.adj) < 0.05 as the cutoff value (19). The identified DEGs were intersected with the CEM-related signatures obtained from the Kyoto Encyclopedia of Genes and Genomes (KEGG) database (https://www.genome.jp/kegg/), MSigDB database (https://www.gsea-msigdb.org/gsea/msigdb), and Reactome database (https://curator.reactome.org/). The summary of the results identified the CEM-DEGs, which were visualized using a Venn diagram and heatmap generated with the “ggplot2” R package.

#### 2.1.2 Functional annotation and pathway enrichment analysis of CEM-DEGs and protein-protein interaction network construction

The CEM-DEGs-related PPI network was constructed using the STRING database (https://cn.string-db.org/) and visualized via Cytoscape software (version 3.9.0). The “clusterProfiler” R package (20) was employed to perform Gene Ontology (GO)/KEGG enrichment analysis on the CEM-DEGs. In the GO enrichment analysis, three categories were included: biological process (BP), cellular component (CC), and molecular function (MF). The results of GO-BP enrichment analysis and KEGG enrichment analysis (*p*.adj < 0.05) were finally visualized using the “ggplot2” package.

#### 2.1.3 Machine learning for screening key CEM-DEGs

In this study, five machine learning methods—Boruta (“Boruta” package) (21), SVM-RFE (“e1071” package and “caret” package) (22, 23), LASSO (“glmnet” package) (24), GBM (“gbm” package) (25), and Xgboost (“xgboost” package) (26)—were employed to identify and filter the important features. Among these algorithms, 10-fold cross-validation was employed to assess the performance of the machine learning models. The final results were ranked according to the importance of the identified results. The intersection of the top 10 ranked results of each algorithm was selected as the key CEM-DEGs for subsequent analysis.

#### 2.1.4 Predictive model construction

Firstly, based on the key CEM-DEGs identified through machine learning, differences in expression levels across various COPD GOLD grades were examined using the dataset GSE47460 (1). Next, lung tissue samples, airway brush samples, induced sputum samples, and peripheral blood samples were collected from patients with chronic obstructive pulmonary disease (COPD) for the construction of predictive models. The lung tissue sample data were sourced from GSE47460 and GSE57148, as elaborated above. Four transcriptome sequencing datasets were incorporated for airway brush samples, namely GSE5058, GSE10006, GSE11784, and GSE20257, encompassing a total of 109 samples (32 COPD samples and 77 control samples) (27–30). The induced sputum sample data were obtained from GSE148004, with a total of 16 samples (7 COPD samples and 9 control samples) (31). The peripheral blood samples were derived from GSE112811, consisting of 42 samples (20 COPD samples and 22 control samples). The processing of these datasets followed the same procedure as described in section 2.1.1 of the Methods. Specific dataset information can be found in **Supplementary Material 1**; **Supplementary Table S1**.

The COPD risk prediction model was constructed utilizing a multivariate logistic regression approach. The model was visualized using a nomogram and fitted using the “lrm” function from the “rms” R package (https://cran.r-project.org/web/packages/rms/index.html). The contribution of each gene to the model was quantified through a Points scale, with the total score (Total Points) mapped to the probability of COPD risk (ranging from 0.1 to 0.9) via weighted summation. The calibration accuracy of the model was assessed using the Bootstrap method, with 500 resampling iterations (B = 500). Consistency between the predicted probabilities and the actual observed probabilities was calculated using the “calibrate” function. The calibration curve was plotted utilizing the “ggplot2” package, where the diagonal line represented the ideal state of calibration. Additionally, the area under the curve (AUC) value of the model was computed using the “pROC” package to evaluate the predictive capability of the gene combination for the disease (32). Finally, the clinical net benefit of the model was analyzed using the “rmda” package, establishing a risk threshold range of 0 to 50% (33). The standardized net benefit of the nomogram model was compared against the “intervention all” (All) and “no intervention” (None) strategies through curve comparison.

#### 2.1.5 GSEA analysis and immune infiltration analysis

In this study, GSEA enrichment analysis was performed by loading R packages such as “clusterProfiler” and “org.Hs.eg.db”. The correlation between the target genes and other genes was evaluated using the Spearman correlation test, and the hallmark gene set files (“h.all.v2024.1.Hs.entrez.gmt”) were utilized in conjunction with the GSEA function for gene enrichment analysis. The final results were visualized by plotting the enrichment map of specific pathways using the “gseaplot2” function. In addition, the results from multiple pathways were compared and visualized to enhance the clarity and intuitiveness of the analysis.

The classification and quantification of immune cells in the merged matrix were based on the expression profiles of 22 immune cell types, which were calculated using the “CIBERSORT” package (34). The final results were visualized as box plots. Then, the relationships between key CEM-DEGs and immune cells were evaluated using Pearson’s correlation analysis, and the resulting data were presented in bubble plots.

#### 2.1.6 Single-cell RNA sequencing analysis of key CEM-DEGs and their involvement in cell-cell communication

Raw expression matrices of lung tissue single-cell RNA-seq data comprising 5 COPD and 3 non-COPD were sourced from the GEO database under accession number GSE173896 (35). Among them, 12,692 single cells derived from non-COPD and 15569 single cells derived from COPD. Then, the integrated raw data were processed using the “Seurat” R package for cell type annotation and subsequent downstream analysis (36). Initially, raw data were filtered to retain genes expressed in more than 200 cells and fewer than 5000 cells, while ensuring that mitochondrial gene expression constituted less than 15% of the total expressed genes. Following normalization, the data from the three grouped samples were combined using the “FindVariableFeatures” function with the “vst” method (nfeatures = 4000). The “IntegrateData” function was then applied to mitigate batch effects and ensure data integrity. In the meantime, the number of principal components in the “RunPCA” function was set to 30. Dimensionality reduction was performed using the “RunUMAP” function. Clustering of the diverse cell groups was achieved through the “FindNeighbors” and “FindClusters” functions, with the resolution parameter set to one.

For each identified cluster, marker genes conserved across genotypes were determined using the “FindMarkers” function. The clusters were subsequently annotated into distinct cell types by referencing established marker genes reported in previous studies and the CellMarker 2.0 database (http://117.50.127.228/CellMarker/), and the distribution of key CEM-DEGs across cell types was visualized using FeaturePlot (37). Next, the relationship between the key CEM-DEGs and various cell clusters was analyzed using Pearson’s correlation test. Based on the expression levels of key CEM-DEGs and the “AddModuleScore_UCell” function in the “UCell” package (38), the cell energy scores related to key CEM-DEGs were calculated. The final results were visualized as featureplot and box plot respectively. It is worth noting that, to further clarify the specific contributing factors of the Ucell score, in this study, we also conducted subpopulation analysis and annotation of epithelial cells and myeloid cells, and determined the specific distribution of the Ucell score.

The calculation of DEGs for each cell cluster between the control group and the COPD group in the single-cell dataset relies on the “FindMarkers” function, with the screening criteria for DEGs being |log2 fold change (FC)| > 0.5 and *p*.adj < 0.05. The results were presented in the form of a volcano plot using the “scRNAtoolVis” package (https://github.com/junjunlab/scRNAtoolVis). Then, the specific cells were reclassified into high-expression and low-expression groups based on the median expression level of the validated key CEM-DEGs, and DEGs between the two groups was calculated for GO/KEGG enrichment analysis.

To analyze intercellular communication, the “CellChat” R package with default parameters was employed to infer potential signaling interactions between cells using a predefined ligand-receptor pair database (39). The cell communication model was constructed using the “createCellChat” function, focusing specifically on interactions between epithelial subtypes and other cell types and highlighting the relationships between cells and the specific ligand-receptor pairs involved in these interactions. The results were visualized using circular plots, heatmaps, and bubble charts to effectively convey the underlying patterns and relationships.

### 2.2 Biology experiment

#### 2.2.1 COPD model *in vivo*

The experimental animal samples were derived from the COPD mouse model previously established by our research group. Six-week-old female C57BL/6 mice were exposed to cigarette smoke for 24 weeks (six days a week, twice a day, each exposure lasting 120 minutes; specific details of the smoke exposure are as follows: tar: 10 mg, nicotine: 0.8 mg, carbon monoxide: 11 mg), while the control group mice were exposed to normal air. For specific information, please refer to the previous study (40).

#### 2.2.2 Cell culture and CSE-induced cell injury model

The human bronchial epithelial cell line BEAS-2B, purchased from Pricella Biotech Technology Co., Ltd. in Wuhan, China, was cultured in high-glucose DMEM supplemented with 1% antibiotics and 10% fetal bovine serum. The cultures were maintained in a humidified environment at 37 °C with 5% CO_2_. Detailed cell culture protocols can be found in previous studies (41).

The cigarette smoke extract (CSE) was utilized to stimulate the human lung epithelial cell line BEAS-2B to construct a cell model. Cells were harvested 24 hours post-treatment. CSE was prepared following a previously reported method, with modifications (42). Specifically, the smoke generated from the complete combustion of five cigarettes was dissolved in 10 ml of DMEM culture medium. Subsequently, the solution was filtered through a 0.22 μm filter, and the optical density (OD) of the stock solution was adjusted to 4.0 using a spectrophotometer, yielding the CSE stock solution for subsequent experiments.

#### 2.2.3 Cell transfection

BEAS-2B cells were grown in T25 cell culture flasks until the density reached approximately 90%, then subcultured into six-well plates, with about 1.2×10^5^ cells per well. After 24 hours, the cells grew to about 60%-70%. According to the kit instructions, 250 μl of siRNA and lipofectamine RNAiMAX (Thermo Fisher Scientific, USA) mixed solution was added to each well. After overnight incubation of the siRNA-liposome complex with the cells, fresh medium was added to continue culturing the cells. The obtained gene knockdown cells were used for subsequent experiments.

#### 2.2.4 Immunohistochemistry staining

The lung tissues of mice were fixed with 4% paraformaldehyde, followed by dehydration, paraffin embedding, sectioning and baking. The obtained tissue sections were dehydrated with xylene and gradient ethanol (100%, 95%, 80%), and then immersed in EDTA antigen retrieval solution for antigen retrieval (high heat for 6 minutes, low heat for 15 minutes). After that, the tissue sections were successively subjected to endogenous peroxidase blocking (treated with 3% H_2_O_2_ for 10 minutes), and blocking (incubated with 5% BSA solution for 1 hour). After the treatment was completed, the tissue sections were incubated with primary antibody (PLA2G1B antibody (15843-1-AP, Proteintech) diluted at 1/200 at 4 °C overnight), and then with secondary antibody (goat anti-rabbit IgG polymer (ZSGB-BIO, China) for 30 minutes). The DAB staining was observed under a microscope, and after the staining was completed, the sections were stained with hematoxylin, differentiated with alcohol hydrochloride, blued, and finally immersed in gradient alcohol and xylene to complete the immunohistochemistry. The immunohistochemistry sections were observed and photographed under a microscope, and the positive area was calculated by ImageJ.

#### 2.2.5 Western blotting

After obtaining the mouse lung tissue, 20 mg of tissue were weighed and cut into small pieces, then 200 μl of RIPA buffer was added and the tissue was homogenized on a tissue homogenizer. The homogenate was then incubated on ice for 10 minutes. For the extraction of cell proteins, 100 μl of RIPA buffer was added to each well of a six-well plate and incubated on ice for 10 minutes. The protein lysates from the mouse lung tissue/cells were collected and centrifuged at 4 °C (12,000 rpm for 10 minutes). The supernatant was collected as the protein sample and could be used for subsequent Western blotting experiments. The protein samples were quantified using a BSA kit, and the loading amount of each sample was adjusted to 20 μg and loaded into 12.5% SDS-PAGE gel lanes for electrophoresis. The proteins were transferred onto PVDF membranes and then subjected to blocking (5% BSA at room temperature for 1 hour), overnight incubation with primary antibodies at 4 °C, and 1-hour incubation with secondary antibodies at room temperature. Finally, the PVDF membranes were exposed using ECL ultra-sensitive luminescent solution. The WB results were processed and quantified using ImageJ. Primary antibodies used were as follows: rabbit anti-PLA2G1B (1/1000, 15843-1-AP, proteintech), rabbit anti-β-actin (1/10000, 81115-1-RR, proteintech), rabbit anti-bax (1/1000, 50599-2-Ig, proteintech), rabbit anti-bcl-2 (1/1000, T40056S, abmart), rabbit anti-caspase3 (1/1000, T40044S, abmart), rabbit anti-cleaved-caspase3 (1/1000, TA7022M, abmart).

#### 2.2.6 Cell counting kit-8 assay

BEAS-2B cells were cultured in T25 flasks until they reached 90% confluence and then subcultured into 96-well plates at a density of 5,000 cells per well. Following an overnight incubation, the cells were grouped according to experimental requirements and further incubated for 24 hours. Subsequently, 10 μl of CCK-8 reagent (Beyotime, China) was added to each well containing 100 μl of culture medium. The plates were incubated for an additional hour in a CO_2_ incubator, and the OD value of each well was measured at 450 nm using a microplate reader. Cell viability was calculated based on the obtained OD values.

#### 2.2.7 RNA extraction and quantitative reverse transcription polymerase chain reaction

Lung tissue and Cells in different groups were subjected to RNA extraction according to the manufacturer’s instructions (Fastagen, China). Subsequently, 1 μg of total RNA was reverse transcribed into cDNA following the kit protocol (Vazyme, China). RT-qPCR was carried out using SYBR Green, and the thermal cycling program was configured as specified in the kit instructions (Vazyme, China). The relative mRNA expression levels were determined using the 2−ΔΔCt method, with ACTB serving as the internal control. All primers were synthesized and provided by Tsingke Biotechnology Company (Beijing, China), and the primer sequences were available in **Supplementary Table S2** of the **Supplementary Materials 1**.

#### 2.2.8 Cell proliferation level detection -Edu assay

The cells in different groups within the 6-well plate were treated according to the protocol provided in the Edu kit (Beyotime, China). Specifically, after adding the Edu solution to the wells and incubating at 37 °C for 2 hours, the cells were gently washed with PBS. Subsequently, the cells underwent fixation and permeabilization sequentially as specified in the kit instructions. Following this, the Click reaction solution was added, and the cells were incubated for 30 minutes. Nuclear staining was then performed using Hoechst 33342. Finally, the results were visualized and imaged under a fluorescence microscope, and the fluorescence intensity was quantitatively analyzed using ImageJ software.

#### 2.2.9 Reactive oxygen species detection

According to the ROS detection kit instructions (Beyotime, China), the treated cells in the 6-well plates were incubated with the DCFH-DA probe at a concentration of 10 μM for 20 minutes at 37 °C. Subsequently, fluorescence images were captured using a fluorescence microscope, and the corresponding fluorescence intensity was quantified using ImageJ software.

### 2.3 Statistical analysis

Statistical analysis was performed using GraphPad Prism (version 8.0), with data presented as mean ± standard deviation. Differences between groups were assessed using one-way ANOVA and student’s t-test. Significance levels were indicated as follows: * *p* < 0.05, ** *p* < 0.01, *** *p* < 0.001, and ns for not statistically significant.

## 3 Results

### 3.1 Screening of the CEM-DEGs and enrichment analysis

**Figure 2:**
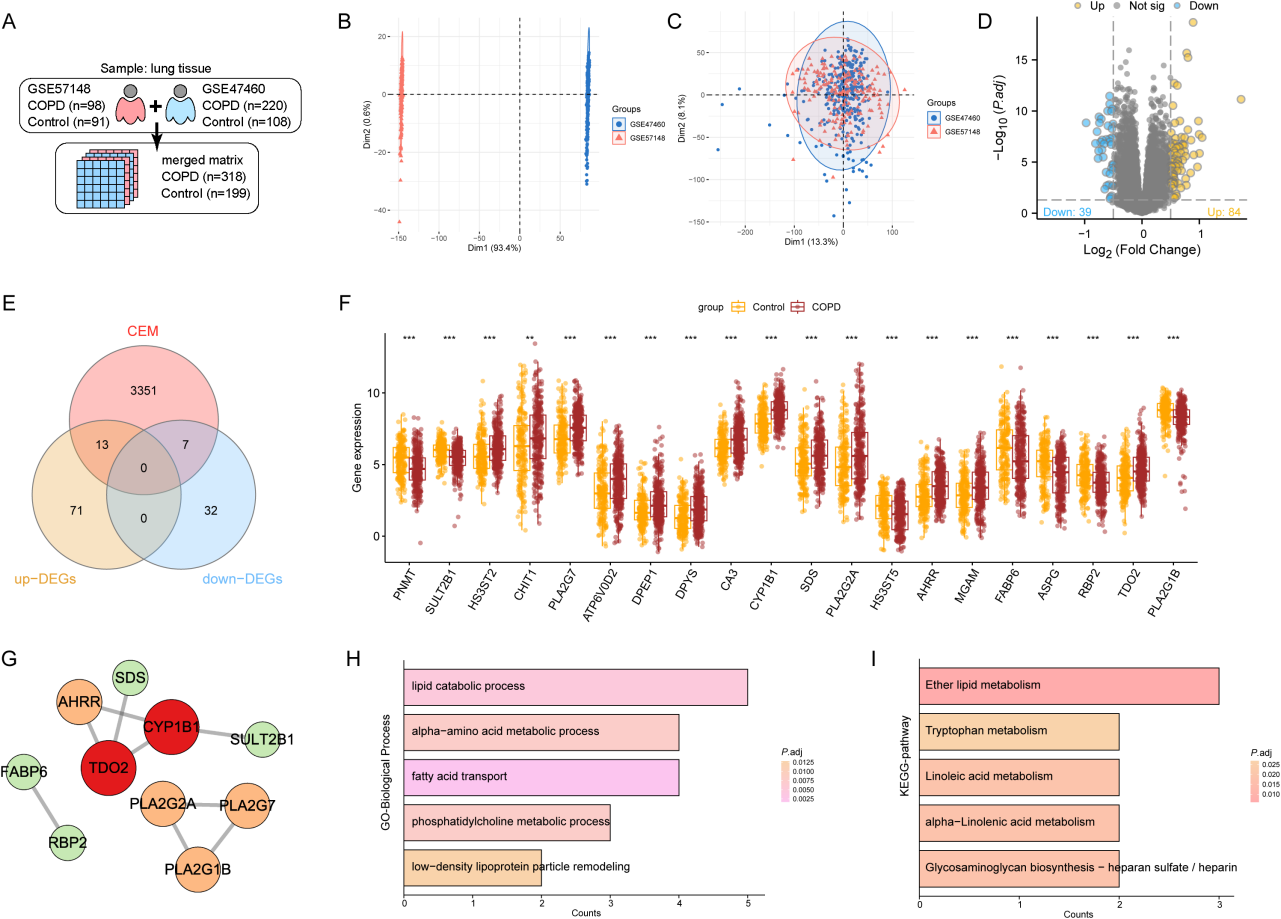
Screening of the CEM-DEGs and enrichment analysis. **(A)** Introduction to dataset information. **(B, C)** PCA analysis results of data distribution before and after batch removal and merging of the dataset. **(D)** Volcano plot of DEGs between the control group and the COPD group. **(E)** Venn diagram showing that among the 123 DEGs, there were 13 upregulated CEM-DEGs and seven downregulated CEM-DEGs. **(F)** The relative expression levels of 20 CEM-DEGs are shown in a bar chart (**p < 0.01, ***p < 0.001). **(G)** PPI network showing the interaction relationships among the 20 CEM-DEGs. **(H, I)** GO/KEGG enrichment results of the 20 CEM-DEGs, presenting the top five enrichment analysis results.

The datasets GSE57148 and GSE47460 were batch-corrected and merged into a comprehensive dataset containing 517 samples (**Figures 2A-C**). By analyzing the DEGs between the control group and the COPD group, it was found that 13 CEM-DEGs were significantly upregulated and seven CEM-DEGs were significantly downregulated (**Figures 2D-F**; **Supplementary Table S3**). A PPI network was constructed for the proteins related to these 20 CEM-DEGs, and the results showed that PLA2G2A and PLA2G7 had the highest combined score (**Figure 2G**; **Supplementary Table S4**). Additionally, GO/KEGG enrichment analysis revealed that these 20 CEM-DEGs were mainly involved in regulating lipid metabolism and amino acid metabolism, such as lipid catabolic process, alpha-amino acid metabolic process, and fatty acid transport in the GO-BP entries, and Ether lipid metabolism, Tryptophan metabolism, and Linoleic acid metabolism in the KEGG enrichment results (**Figures 2H, I**; **Supplementary Table S5**).

### 3.2 Machine learning identified the key CEM-DEGs and the predictive model construction

**Figure 3:**
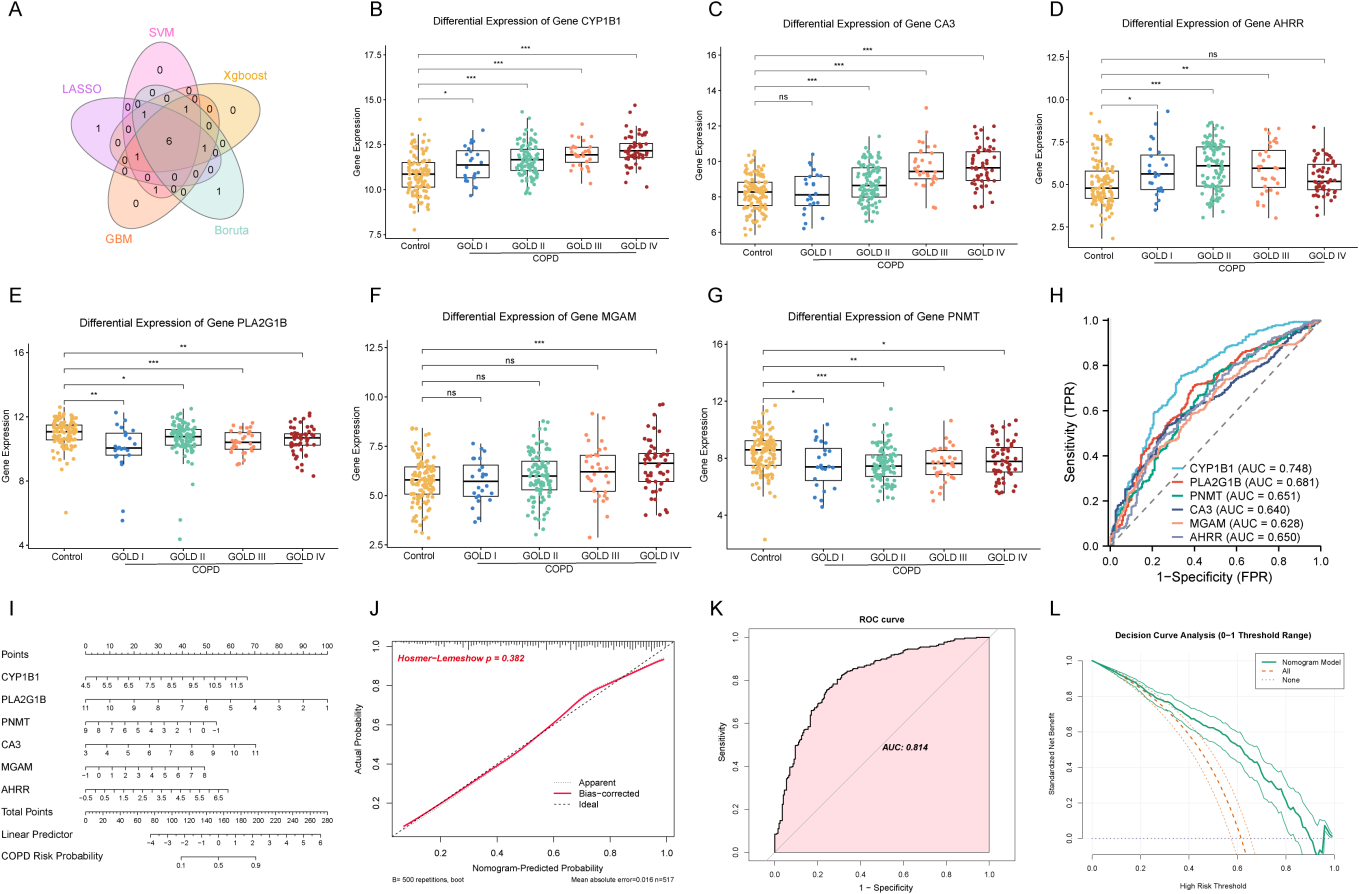
Machine learning identified the key CEM-DEGs and the nomogram model construction. **(A)** Venn plot of the intersection of the screening results of five machine learning algorithms. **(B-G)** The relative expression levels of six key CEM-DEGs in different GOLD grades (*p < 0.05, **p < 0.01, ***p < 0.001, and “ns” represented not significant). **(H)** ROC curve of the diagnostic efficiency for six key CEM-DEGs. **(I)** The nomogram model construction. **(J)** Calibration curve of the constructed predictive model. **(K)** ROC curve of the diagnostic efficiency for nomogram model. **(L)** Evaluate the clinical utility of predictive models using the DCA decision curve.

To determine which of the 20 CEM-DEGs are the main regulatory genes, five machine learning algorithms were used in this study to screen out the key variables in each model, and the results of the five machine learning methods were summarized to obtain six key CEM-DEGs (**Supplementary Figures S1A-E**; **Figure 3A**). Based on the sample information obtained from GSE47460 and the COPD grades according to the GOLD guidelines (1), this study further analyzed the association between these six key CEM-DEGs and COPD grades. The results showed that the expression levels of cytochrome P450 family 1 subfamily B member 1 (CYPIB1) and carbonic anhydrase 3 (CA3) significantly increased with the increase of COPD classification. The expression level of aryl hydrocarbon receptor repressor (AHRR) significantly increased in GOLD I, II, and III, but there was no significant change compared with the control group in GOLD IV; PLA2G1B and phenylethanolamine N-Methyltransferase (PNMT) were significantly downregulated in all four stages, but no correlation with disease severity was observed; maltase-glucoamylase (MGAM) was significantly increased only in GOLD IV, the most severe case of COPD (**Figures 3B-G**). The ROC curve showed that the single-gene diagnostic efficiency of these six key CEM-DEGs was low (AUC < 0.8) (**Figure 3H**). Therefore, a nomogram model was further constructed in this study for COPD risk prediction, and the total score of the six CEM-DEGs in the prediction model was used to map the final risk probability, with *PLA2G1B* being the main contributing marker (**Figure 3I**). Additionally, the p-value of the Hosmer-Lemeshow (HL) test in the calibration curve was 0.382, indicating no significant difference between the predicted and actual values, and the error between the actual and predicted disease risks was minimal (**Figure 3J**); the AUC value in the ROC curve was 0.814, indicating that the model had good discrimination ability (**Figure 3K**); the DCA curve results showed that the net benefit of this nomogram was higher than that of the positive and negative controls, and its net benefit was higher than that of using any single biomarker alone, thus demonstrating significant clinical utility (**Figure 3L**).

**Figure 4:**
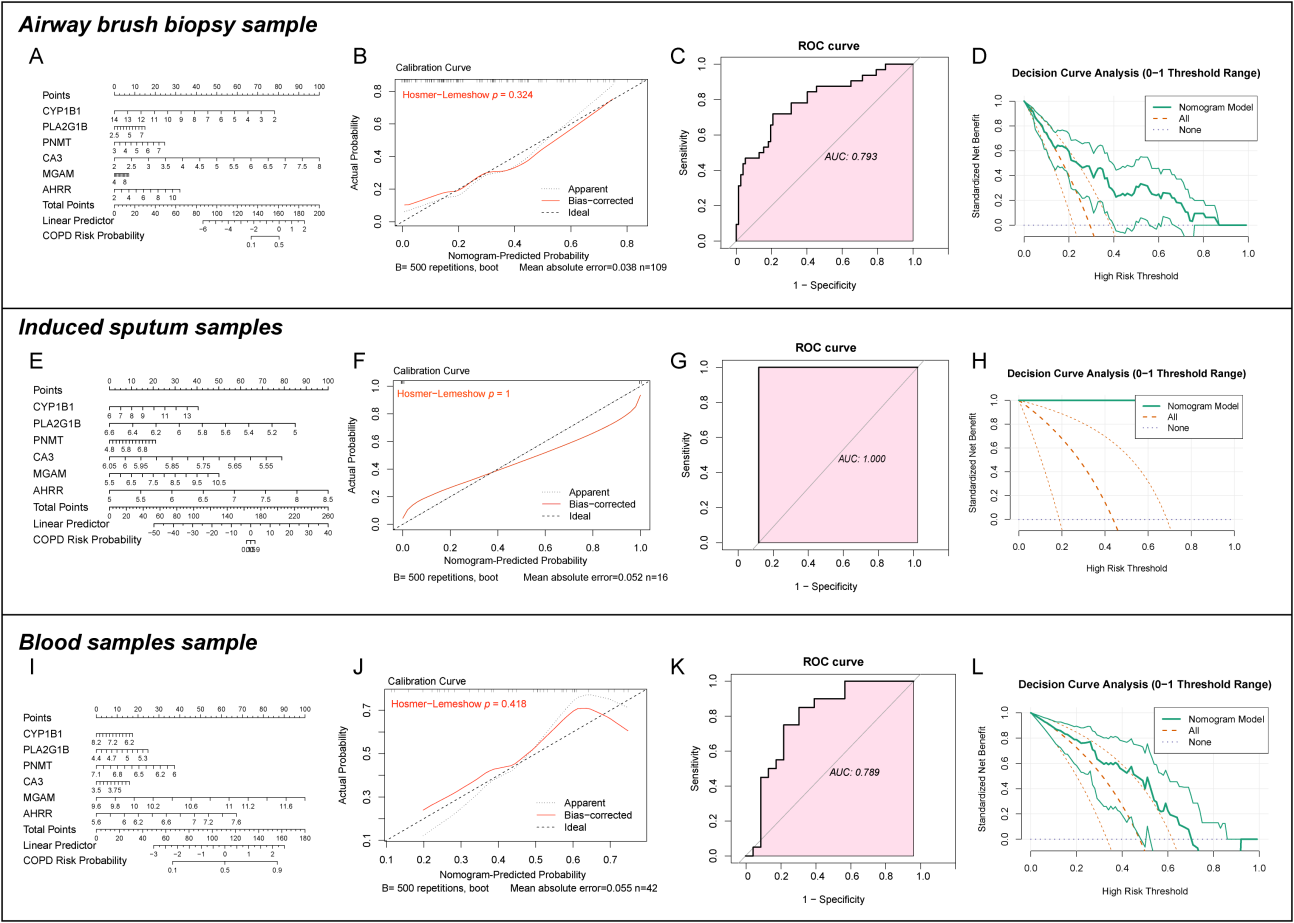
Construct COPD-related nomogram in different samples. **(A-D)** constructed COPD prediction models based on sequencing data from airway brushings in datasets GSE5058, GSE10006, GSE11784, and GSE20257, where **(A)** represents the nomogram, **(B)** the calibration curve, **(C)** the ROC curve, and **(D)** the DCA curve. **(E-H)** present COPD prediction models derived from induced sputum samples (GSE148004) using six CEM-DEGs, with **(E)** as the nomogram, **(F)** the calibration curve, **(G)** the ROC curve, and **(H)** the DCA curve. **(I-L)** show COPD prediction models developed from peripheral blood samples (GSE112811) using the same six CEM-DEGs, where **(I)** denotes the nomogram, **(J)** the calibration curve, **(K)** the ROC curve, and **(L)** the DCA curve.

To evaluate the broader clinical applicability of the identified CEM-DEGs, this study further utilized transcriptome sequencing datasets from airway brush, induced sputum, and peripheral blood samples of COPD patients to construct and validate predictive models. In the model derived from airway brush samples, CA3 was the primary contributing factor, yielding an AUC of 0.793 with good predictive performance (HL test p = 0.324) (**Figures 4A–D**). For the induced sputum-based model, AHRR emerged as the dominant predictor, achieving a perfect AUC of 1.0, indicating excellent predictive accuracy (HL test p = 1.0) (**Figures 4E–H**). In the peripheral blood-derived model, MGAM was the main contributor, with an AUC of 0.789, also demonstrating strong predictive capability (HL test p = 0.418) (**Figures 4I–L**). These findings collectively suggest that the six CEM-DEGs exhibit robust diagnostic and predictive potential for chronic obstructive pulmonary disease across multiple sample types and biological contexts.

### 3.3 Potential functions of six key CEM-DEGs and associated immune cells

**Figure 5:**
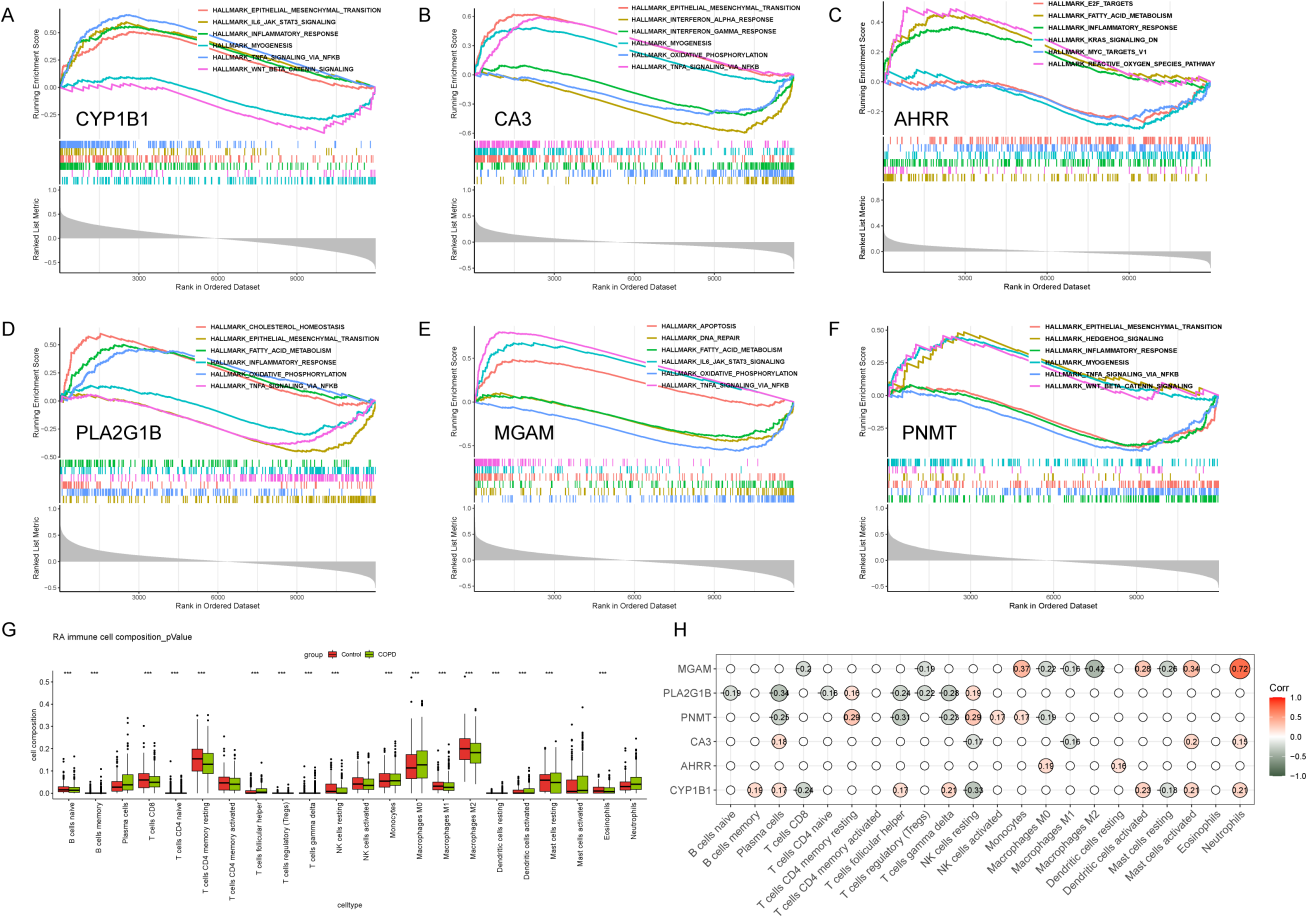
Analysis of the potential functions and immune cell correlations of six key CEM-DEGs. **(A-F)** GSEA analysis of the potential functions of six key CEM-DEGs (The overall upward peaks in the figure (enrichment score greater than 0) indicate activation, while the overall downward peaks (enrichment score less than 0) indicate inhibition). **(G)** Immune infiltration analysis of the composition of 22 types of immune cells in the merged dataset (***p < 0.001). **(H)** The correlation analysis results between six CEM-DEGs and 22 types of immune cells are shown. Only the results with a p-value less than 0.05 are displayed in the bubble chart.

Based on public databases, this study collated and collected the basic information of six key CEM-DEGs. For details, please refer to **Supplementary Table S6** in the **Supplementary Material 1**. When performing GSEA enrichment analysis on the six key CEM-DEGs to identify their associated functions, the results indicated that all these genes were involved in regulating inflammatory responses (**Figures 5A-F**, **Supplementary Table S7**). Except for *AHRR*, the other five genes (*CYP1B1*, *CA3*, *PLA2G1B*, *MGAM*, *PNMT*) were all involved in regulating the TNFA signaling pathway, among which *CYP1B1* and *MGAM* were also involved in the IL6-JAK-STAT3 pathway. The enrichment results of *AHRR* were mainly related to various metabolic pathways, such as fatty acid metabolism and cholesterol homeostasis (**Figure 5C**, **Supplementary Table S7**). Additionally, it is worth noting that these genes were also closely associated with epithelial-mesenchymal transition and oxidative phosphorylation pathways, collectively indicating that these genes play a significant role in the crosstalk between metabolism, inflammatory responses, and oxidative stress. Immune infiltration analysis was conducted on the merged dataset, and the results showed that the most abundant cell type was macrophages, followed by T cells (**Figure 5G**). Further correlation analysis revealed a close association between six key CEM-DEGs and immune regulation, especially *MGAM*, which had the highest correlation with neutrophils (*R* = 0.72, *p* < 0.05) (**Figure 5H**).

### 3.4 Analysis of the expression level of six key CEM-DEGs based on single-cell data

**Figure 6:**
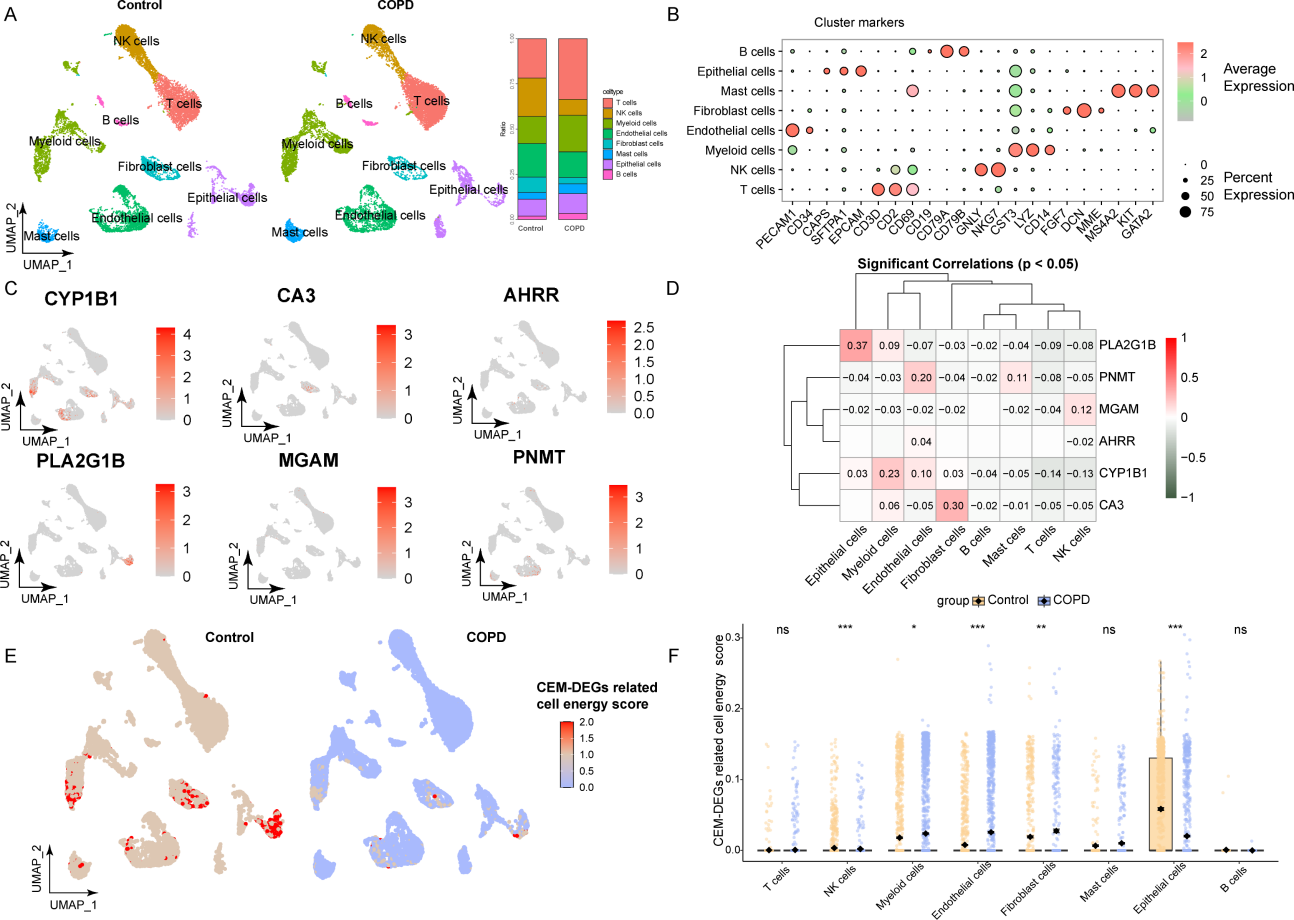
Analysis of six key CEM-DEGs based on single-cell transcriptome datasets. **(A)** The distribution and proportion of different cell types in the control group and the COPD group. **(B)** The distribution of marker expression among different cell types in bubble chart. **(C)** The relative expression levels of six key CEM-DEGs in different cell types. **(D)** The correlation results between six key CEM-DEGs and different cell types (Only results with *p* < 0.05 are presented). **(E)** The UCell cell energy score constructed based on 6 key CEM-DEGs in UMAP plot. **(F)** Analysis of the differences in cell energy scores among different cell types in different groups (ns p > 0.05 (not statistically significant), * *p* < 0.05, ** *p* < 0.01, *** *p* < 0.001).

In this single-cell sequencing dataset, cell types were labeled by different markers (For T cells, the gene markers included *CD3D*, *CD2* and *CD69*; B cells were identified by *CD19*, *CD79A* and *CD79B*; NK cells expressed *NKG7* and *GNLY*; myeloid cells were distinguished by the presence of *CD14*, *LYZ* and *CST3*; while endothelial cells exhibited expression of *CD34* and *PECAM1*. Additionally, fibroblast cells were marked by *MME*, *DCN* and *FGF7*; mast cells by *GATA2*, *KIT* and *MS4A2*; and epithelial cells by *EPCAM*, *SFTPA1* and *CAPS*), and a total of 8 types of cells were identified, including epithelial cells, endothelial cells, myeloid cells, T cells, NK cells, fibroblasts, B cells and mast cells(**Figures 6A, B**) (43, 44). By analyzing the expression levels and cell distribution of six key CEM-DEGs, the results showed that *CYP1B1* was most highly correlated with myeloid cells, *CA3* with fibroblasts, *PLA2G1B* with epithelial cells, *MGAM* with NK cells, *PNMT* with endothelial cells, while *AHRR* had no high correlation with the above eight types of cells (**Figures 6C, D**). Furthermore, a cell energy score was constructed based on these six key CEM-DEGs. The results showed that there was a significant difference in the cell energy score between the COPD group and the control group, indicating that there were certain cell energy metabolism alterations in the COPD group (**Figure 6E**, **Supplementary Table S8**). Notably, the highest cell energy score was found in the epithelial cells of the control group, and the most significant difference was observed in the COPD group (**Figure 6F**).

**Figure 7:**
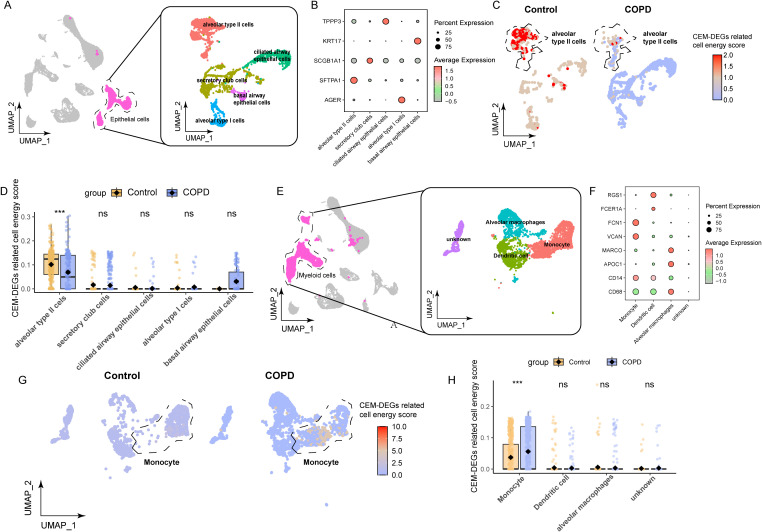
Subpopulation analysis of epithelial cells and myeloid cells based on cell energy score. **(A)** The UMAP plot illustrates the distribution of subpopulations among the extracted epithelial cells. **(B)** The bubble chart displays the marker genes specific to each epithelial cell subpopulation. **(C–D)** The distribution of UCell scores across different epithelial cell subpopulations and statistical comparisons between groups are shown. **(E)** The UMAP plot depicts the subpopulation structure of the isolated macrophages. **(F)** The bubble chart presents the characteristic markers for distinct macrophage subtypes. **(G–H)** The UCell score distribution among macrophage subgroups and the results of intergroup statistical analyses are displayed (ns *p* > 0.05 (not statistically significant), *** *p* < 0.001).

Given the elevated cellular energy scores observed in both epithelial cell and myeloid cell populations, along with the substantial heterogeneity within these cell populations, we carried out subsequent subpopulation analyses. Initially, in the case of epithelial cells, based on distinct markers, the epithelial cells were classified into subpopulations, namely alveolar type I epithelial cells (AGER^+^), alveolar type II epithelial cells (SFTPA1^+^), goblet cells (SCGB1A1^+^), ciliated cells (TPPP3^+^), and basal cells (KRT17^+^) (**Figures 7A, B**). Cellular energy score analysis revealed that the alveolar type II (ATII) cells were the predominantly affected cell population. Specifically, the scores of ATII cells were significantly lower in the chronic obstructive pulmonary disease (COPD) group compared to the control group (**Figures 7C, D**). Regarding myeloid cells, they could be primarily divided into three groups: the monocyte population (FCN1^+^VCAN^+^), the dendritic cell population (RGS1^+^FCER1A^+^), and the alveolar macrophages population (MARCO^+^APOC1^+^) (**Figures 7E, F**). Among these, monocytes were the main high-scoring cell population. Intriguingly, in contrast to epithelial cells, the scores of monocytes were higher in the COPD group than in the control group (**Figures 7G, H**).

### 3.5 The influence of the expression level of PLA2G1B on the function of epithelial cells and cell communication

**Figure 8:**
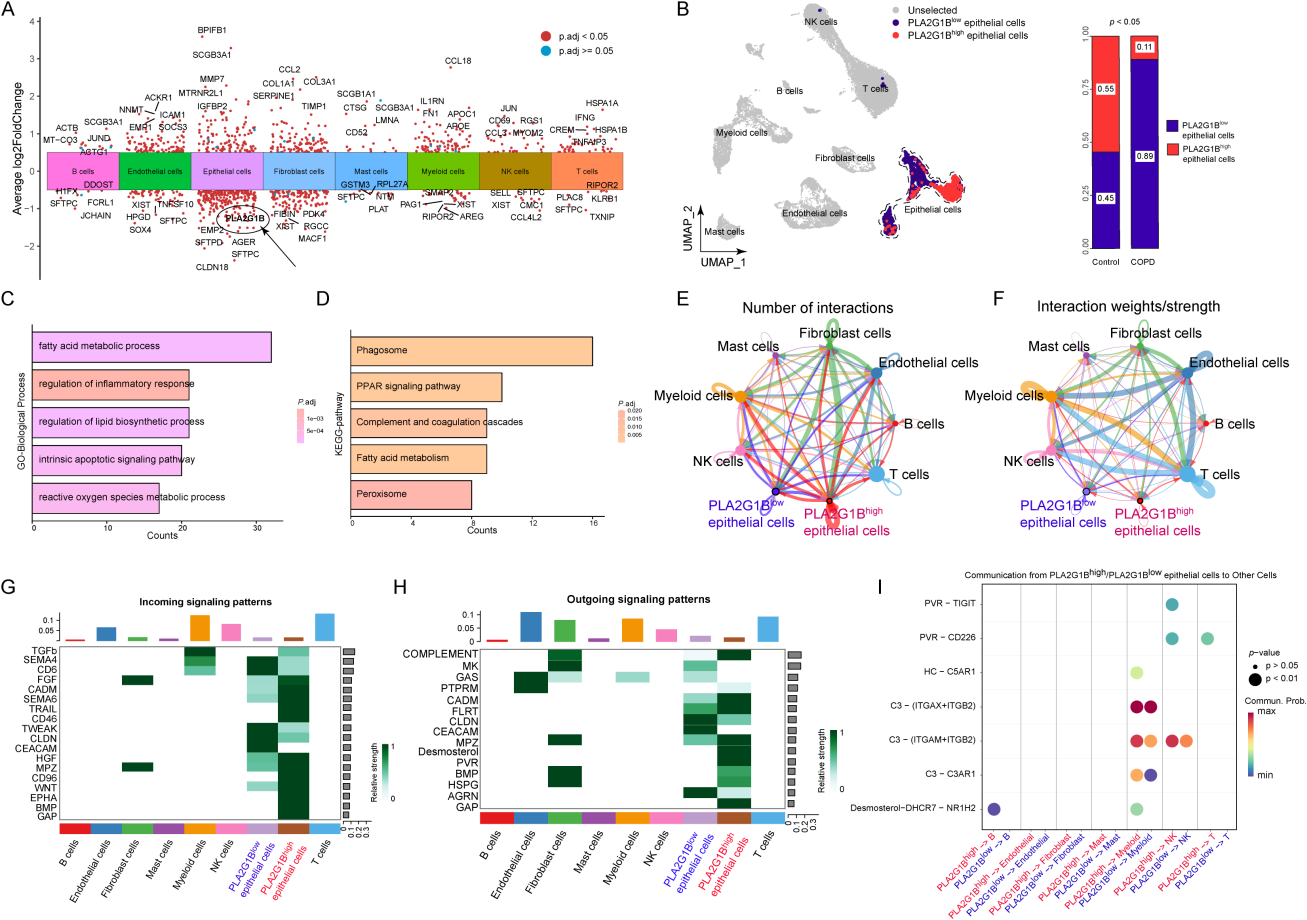
Single-cell level analysis of the phenotypic function and cell communication of PLA2G1B. **(A)** DEGs between the control group and COPD group of different cell types in volcano plot. **(B)** The distribution and proportion of *PLA2G1B* ^high^ epithelial cells and *PLA2G1B* ^low^ epithelial cells. **(C, D)** GO/KEGG enrichment analysis of DEG between *PLA2G1B* ^high^ epithelial cells and *PLA2G1B* ^low^ epithelial cells. **(E-F)** Network Diagram Analysis: Cell Communication Results [**(E)** Number of Communications, **(F)** Communication Weight]. **(G, H)** Heatmap showing the relative intensity of each signaling pathway network in each cell type cluster. [**(G)** plot showing the incoming signal patterns, and **(H)** plot showing the outgoing signal patterns.] **(I)** Dot plot showing the receptor pairings for signal transduction between *PLA2G1B* ^high^ epithelial cells and *PLA2G1B* ^low^ epithelial cells and other cell types, respectively.

By obtaining the DEGs of different cell types between the control group and the COPD group, the results showed that among the six key CEM-DEGs, *PLA2G1B* was significantly downregulated in the epithelial cell category of the COPD group, which was consistent with the previous transcriptome results (**Figure 8A**, **Supplementary Table S9**). Based on this, the epithelial cells in the single-cell data were divided into *PLA2G1B* ^high^ epithelial cells group and *PLA2G1B* ^low^ epithelial cells group for subsequent phenotypic and cell communication analyses (**Figure 8B**). After re-grouping the epithelial cells, it could be seen that the number of *PLA2G1B* ^low^ epithelial cells in the COPD group was significantly increased compared with the control group (*p* < 0.05) (**Figure 8B**). By identifying the DEGs between the *PLA2G1B* ^high^ epithelial cells and *PLA2G1B* ^low^ epithelial cells and conducting functional analysis, it was found that these genes were involved in regulating lipid metabolism, inflammatory response, apoptosis, oxidative stress, phagosome pathway, PPAR pathway and peroxisome pathway, etc (**Figures 8C-D**; **Supplementary Tables S10-11**). In addition, the cell communication analysis revealed that *PLA2G1B* ^high/low^ epithelial cells have a closer relationship with myeloid cells and T cells, with a greater number of cell communications and a higher proportion of weight (**Figures 8E-F**). For different signaling pathways, *PLA2G1B* ^low^ epithelial cells received more signals from SEMA4, CD6, TWEAK, CLDN, and CEACAM, and the main outgoing signals were CLDN, CEACAM, and ARGN (**Figures 8G-H**). More specifically, the differences in receptor-ligand pairs exist in the cell communication between *PLA2G1B* ^high^ and *PLA2G1B* ^low^ epithelial cells with B cells, myeloid cells, NK cells, and T cells (**Figure 8I**).

### 3.6 PLA2G1B was down-regulated in COPD models and participated in the regulation of inflammation, oxidative stress and cell death

**Figure 9:**
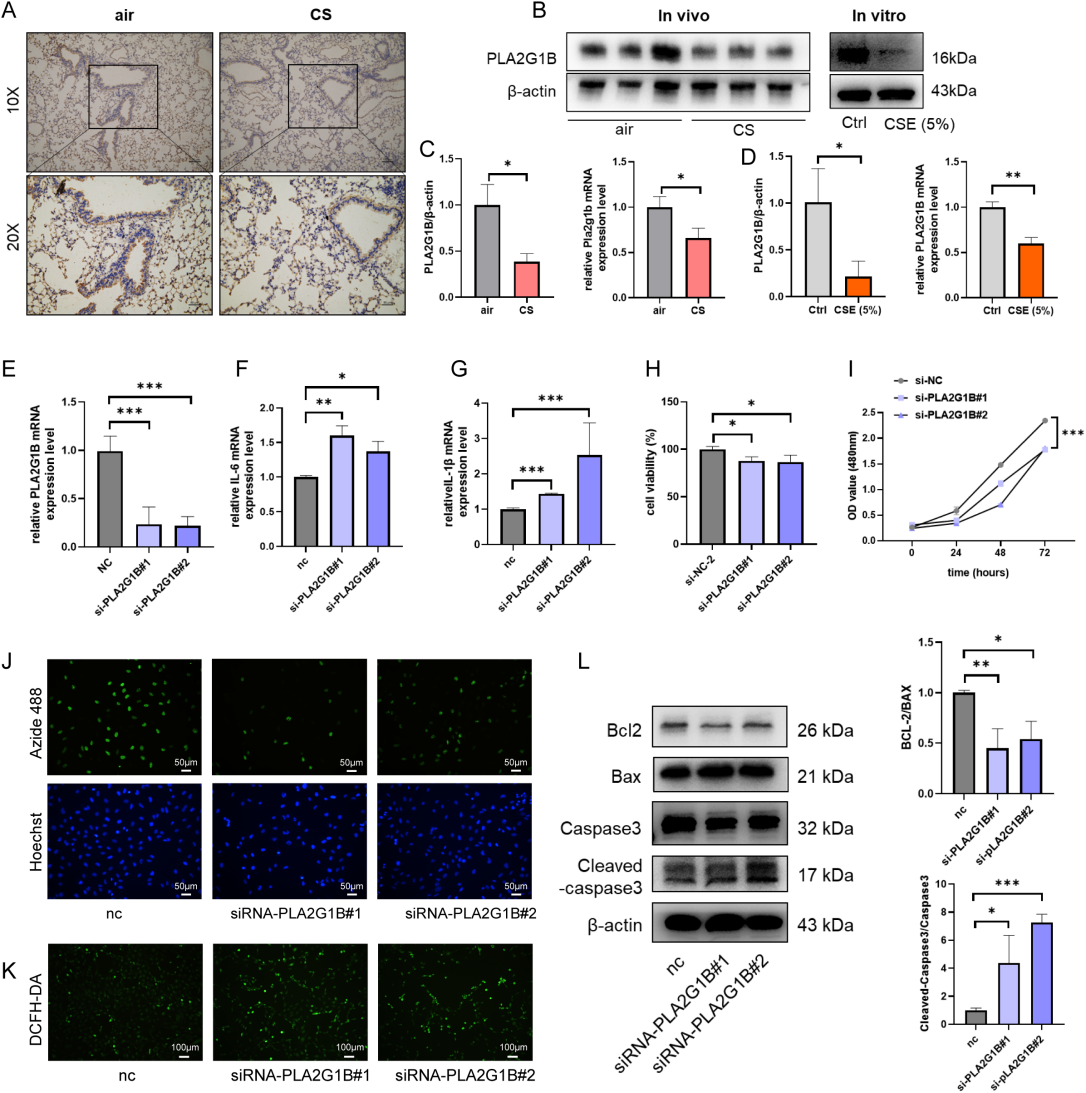
Down-regulated PLA2G1B is involved in regulating inflammation, proliferation, oxidation and apoptosis of epithelial cells. **(A)** Immunohistochemical detection of PLA2G1B expression levels in mouse lung tissue (n=3), showing both 10x basic images and 20x magnified images respectively. **(B-D)** The protein and mRNA expression levels of PLA2G1B in mouse lung tissues (n=3) and BEAS cells, detected by western blotting and qPCR. **(E)** qPCR for detecting the knockdown efficiency of siRNA on the expression level of PLA2G1B in BEAS-2B cell. **(F-G)** The influence of PLA2G1B knockdown on the transcriptional levels of inflammatory factors IL-1β and IL-6. **(H-I)** The CCK8 assay was used for detecting cell activity and measuring the OD values of cells at different time points. **(J)** Detecting cell proliferation ability, in which Azide-488 was used to label the intracellular Edu level and Hoechst was used to label the cell nucleus. **(K)** The intracellular ROS level was detected by labeling intracellular ROS with DCFH-DA. **(L)** The results of western blotting of the protein levels of apoptosis-related markers (BCL2, BAX, Cleaved-caspase3, caspase3) in cells. (Data are shown as mean ± SD (n = 3), *p < 0.05, **p < 0.01, ***p < 0.001).

To further validate the results of bioinformatics analysis, COPD-related animal and cell models were constructed in this study. As PLA2G1B has made a significant contribution to the predictive model of COPD and has been verified in single-cell sequencing data, the subsequent experiments mainly focus on exploring the mechanisms related to PLA2G1B. Firstly, in the mouse model, compared with the control group, the expression level of PLA2G1B in the lung tissue of COPD mice models established by cigarette smoke inhalation for 6 months was significantly downregulated, including both protein and mRNA levels. Notably, immunohistochemical results showed that PLA2G1B was significantly downregulated in the airway epithelial cells of COPD mice, which was consistent with the single-cell analysis results (**Figures 9A-C**). Additionally, in the *in vitro* experiment, compared with the control group, when the epithelial cell line BEAS-2B was stimulated with CSE (5%), the protein and mRNA levels of PLA2G1B were also significantly downregulated (**Figures 9B, D**).

Given that cell death, inflammatory responses, and oxidative stress represent key pathophysiological mechanisms involved in the progression of COPD, and considering that the aforementioned analysis suggested a potential role of PLA2G1B in influencing these phenotypes, this study employed siRNA transfection to knock down PLA2G1B expression in epithelial cells in order to investigate its association with cell death, inflammatory responses, and oxidative stress (**Figure 9E**). The results showed that the knockdown of PLA2G1B led to an increase in the transcriptional levels of inflammatory-related factors (IL-1β, IL-6) in BEAS-2B cells (**Figures 9F, G**). At the same time, the knockdown of PLA2G1B led to a decrease in cell viability, mainly manifested as a decline in proliferation ability. When Edu was used to label proliferating cells, the results showed that the number of proliferating cells in the PLA2G1B knockdown group was significantly lower than that in the control group (**Figures 9H-J**). In addition, when conducting functional enrichment analysis related to PLA2G1B, it was found that PLA2G1B regulated the ROS pathway and apoptosis (**Figure 9C**). Therefore, in this study, the intracellular ROS level was detected using the DCFH-DA probe, and the apoptotic signal was identified by detecting the protein levels of classic apoptotic pathway markers (BCL2/BAX, Cleaved-caspase3/caspase3). The results showed that the knockdown of PLA2G1B led to a significant increase in intracellular ROS levels and was accompanied by the activation of the apoptotic pathway (BCL2/BAX significantly decreased, Cleaved-caspase3/caspase3 significantly increased) (**Figures 9K, L**).

## 4 Discussion

As a kind of chronic respiratory disease, COPD has a high incidence and mortality in the world population, which seriously affects the life and health of middle-aged and elderly people (45). However, the exact mechanism of COPD occurrence is not yet fully understood, especially the molecular mechanism of CEM. Integrating bioinformatics analysis to explore valuable markers and potential therapeutic targets related to CEM disorders will be beneficial for the early diagnosis and drug development of precision treatment of COPD in the future.

In this study, the two largest COPD transcriptome sequencing datasets were integrated for bioinformatics analysis. Multiple machine learning algorithms were used to identify six key CEM-DEGs (including *CYP1B1*, *CA3*, *AHRR*, *MGAM*, *PNMT*, and *PLA2G1B*) from numerous DEGs. The nomogram model and ROC curve were employed to evaluate the diagnostic and predictive capabilities of these genes for COPD. It is worth noting that through the analysis of the predictive effects of these six CEM-DEGs on COPD in different sample types, their advantages in diagnosing COPD have been further confirmed. Besides lung tissue, they are also applicable to more convenient sample types such as peripheral blood, induced sputum, and airway brushings. The GSEA analysis results indicated that these genes were mainly involved in lipid metabolism, inflammation, oxidative phosphorylation, and epithelial-mesenchymal transition, suggesting that the outcome of metabolic dysregulation might be associated with the induction of cellular inflammatory responses, oxidative stress, and airway remodeling. Additionally, recent studies have reported that regulating mitochondrial metabolism and *de novo* NAD synthesis in macrophages could exacerbate inflammatory responses and oxidative stress, and stimulating lipid metabolism in lung epithelial cells could increase oxidative stress and disrupt redox homeostasis. Overall, these findings highlighted the intricate crosstalk between CEM, inflammatory responses, and oxidative stress in lung diseases (11, 16, 46, 47).

Among the six CEM-DEGs, *CYP1B1* regulates fatty acid and steroid hormone metabolism by encoding the cytochrome P450 enzyme superfamily and serves as a therapeutic target for ocular diseases and various tumors (48–50). Toxicological study found that exposure to particulate matter could lead to a significant increase in the expression level of CYP1B1 in epithelial cells (51), which was consistent with the results of this study. In our study, CYP1B1 was mainly upregulated in COPD, especially closely related to myeloid cells and involved in the occurrence of inflammatory responses. Specifically, it participates in the IL-6-JAK-STAT3 signaling and TNF signaling. CAH3, encoded by the *CA3* gene, primarily catalyzes carbon dioxide metabolism within cells. Multi-omics studies have revealed its involvement in the regulation of muscle function and its significant diagnostic value for diseases such as muscle atrophy, hypertrophic cardiomyopathy, and non-alcoholic steatohepatitis (52–54). In this study, it was found that CA3 was highly expressed in patients with moderate to severe COPD, closely associated with the activation of interferon signaling, and was predominantly expressed in fibroblasts in the lungs. AHRR is recognized as a biomarker closely linked to smoking, which significantly increases AHRR methylation levels, thereby impacting cell growth and differentiation, while its regulatory mechanisms in immunity remain largely unexplored (55, 56). In this study, we found that *AHRR* expression was upregulated in the lung tissues of COPD patients, and GSEA analysis indicated that AHRR’s regulation of cell growth and differentiation may be associated with its influence on the KRAS pathway, as well as MYC and E2F signaling. Intriguingly, AHRR could interact with CP1B1 via the Ah receptor pathway, influencing oxidative stress and mediating toxic responses (57). MGAM regulates glucose metabolism by encoding the protein MGA, thereby influencing the digestive and absorptive processes in the human body. Moreover, multiple studies have revealed its diagnostic significance in various diseases through bioinformatics analysis, including bronchopulmonary dysplasia (58), pain-depression comorbidity (59), intervertebral disc degeneration (60), and stroke (61). In this study, we found that MGAM was significantly elevated in severe COPD, not only affecting biological processes such as inflammation and oxidative stress, but also participating in the regulation of apoptosis and DNA repair, thereby influencing the survival of cells. The PNMT protein is a key enzyme that catalyzes the conversion of norepinephrine to epinephrine. It was regulated by glucocorticoids, hypoxia, and the cAMP signaling pathway, and was associated with the occurrence of mood disorders and neuroendocrine tumors (62–64). Furthermore, PNMT could also be involved in neuroendocrine tumors through epigenetic mechanisms (65), highlighting its potential as a therapeutic target for diseases associated with adrenergic signaling dysregulation. In lung tissue, this study revealed that the low expression of PNMT might be closely related to the inflammation associated with COPD, regulating cell proliferation and transformation, etc.

The phospholipase A2 (PLA2) family includes multiple subtypes such as PLA2G1, PLA2G2, PLA2G4, PLA2G6, PLA2G7, and PLA2G16, which exert various biological functions by hydrolyzing the sn-2 position of glycerophospholipids to release fatty acids and lysophospholipids (66). Among them, PLA2G1B was involved in the progression of various diseases, including tumors, ulcerative colitis, and rheumatoid arthritis, etc (67–69). Notably, PLA2G1B, as a double-edged sword, played opposite roles in different diseases, but the specific molecular mechanism remained unclear. In this study, bioinformatics analysis revealed that PLA2G1B was significantly downregulated during the progression of COPD, particularly in the epithelial cell category. The results were also verified in animal and cell models (with significant downregulation at both the transcriptional and protein levels), indicating its association with poor prognosis. Similar to the findings of this study, Guan et al. also identified, through bioinformatics analysis, that low expression levels of PLA2G1B in tumors were significantly associated with reduced survival rates (68). Further enrichment analysis related to the function of PLA2G1B revealed that PLA2G1B not only regulated inflammation, oxidation, and lipid metabolism but also participated in the regulation of apoptosis and cell communication. Some of these results were verified in the cell experiments of this study. In addition, in this study, it was found that the epithelial cells with low/high expression of PLA2G1B mainly interacted with myeloid cells. Particularly, the activation of complement signals in epithelial cells with low expression of PLA2G1B was significantly lower than that in epithelial cells with high expression of PLA2G1B, indicating that it could be involved in immune regulation. In summary, this study elucidated the specific role of PLA2G1B in COPD and its regulatory influence on epithelial cell inflammation, oxidative stress, and apoptosis. These findings expand the current understanding of PLA2G1B mechanisms and its involvement in the pathophysiological processes of COPD, suggesting that targeted overexpression of PLA2G1B in epithelial cells may hold therapeutic potential in mitigating COPD progression.

It was worth noting that this study still had certain limitations that need to be improved in future research. Firstly, the data mining and analysis in this study were conducted using public databases, and the results obtained only remain at the initial theoretical stage, lacking the validation of the predictive results of the related biomarkers’ prognostic models through clinical trials. In addition, future studies should collect BALF samples from COPD patients to enable a more comprehensive and in-depth investigation into the molecular function of PLA2G1B. Secondly, this study only focused on the impact of gene alterations on functional phenotypes and did not further explore the differential changes in related metabolites. Knockdown/overexpression of PLA2G1B at the animal level and collection of lung tissue samples for metabolomics research may provide new insights into the pathogenesis of COPD. In the future, it is possible to explore related small molecule agonist drugs to further expand the treatment options for COPD.

## 5 Conclusions

In this study, through comprehensive analysis of multiple datasets, six key CEM-DEGs (*CYP1B1*, *CA3*, *AHRR*, *MGAM*, *PNMT, PLA2G1B*) were identified, which can be used to construct a COPD prediction model. Furthermore, we confirmed that the downregulated *PLA2G1B* in COPD is associated with the biological processes of inflammation, oxidative stress, and apoptosis in epithelial cells. Overall, this study provided new insights into the molecular mechanism of COPD from the perspective of CEM and offered new strategies for the diagnosis and treatment of COPD.

## Data Availability

The raw data supporting the conclusions of this article will be made available by the authors, without undue reservation.
