# Supplementary material for: Integrating bioinformatics and molecular experiments to reveal the critical role of the cellular energy metabolism-related marker PLA2G1B in COPD epithelial cells
Source: Front Immunol. 2025 Oct 16;16:1666195. doi: 10.3389/fimmu.2025.1666195 (PMC12571731; doi:10.3389/fimmu.2025.1666195)
Supplement: Supplementary file 2 [file SupplementaryFile1.docx]

Supplementary Material 2


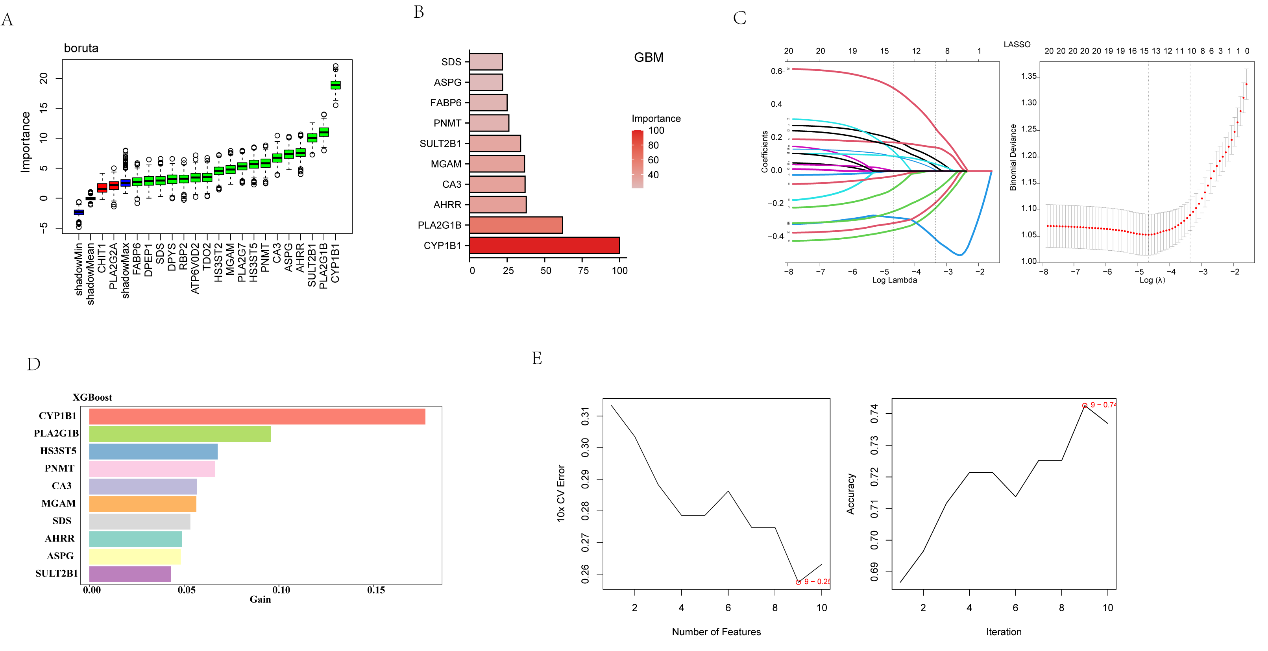


**Fig. S1** Five machine learning methods for identifying key MR-DEGs **A** Boruta algorithm **B** GBM algorithm **C** LASSO algorithm **D** Xgboost algorithm **E** SVM-RFE algorithm.
